# Correlation between cardiorespiratory fitness and body composition in individuals with different glucose metabolism statuses

**DOI:** 10.3389/fendo.2026.1726184

**Published:** 2026-05-29

**Authors:** Binbin Liu, Junliang Li, Ziru Niu, Qiang Lu

**Affiliations:** 1 Department of Functional Examination, First Hospital of Qinhuangdao, Hebei, Qinhuangdao, China; 2 Department of Inspection Centres, First Hospital of Qinhuangdao, Hebei, Qinhuangdao, China; 3 Department of Endocrinology, First Hospital of Qinhuangdao, Hebei, Qinhuangdao, China

**Keywords:** body composition, cardiorespiratory fitness, diabetes mellitus, skeletal muscle, visceral fat area

## Abstract

**Objective:**

To investigate Cardiorespiratory Fitness indicators in populations with different glucose metabolism statuses and analyze their correlation with body composition.

**Methods:**

This study retrospectively included 144 individuals who voluntarily underwent cardiopulmonary exercise testing at Qinhuangdao First Hospital from January 2023 to June 2025. Based on oral glucose tolerance test (OGTT) results, participants were categorized into the Normal Glucose Tolerance Group (n = 76), Prediabetes Group (n = 22), and Diabetes Mellitus Group (n = 46). Differences in general characteristics, laboratory indicators, Cardiorespiratory Fitness, and body composition were analyzed among the three groups. Univariate correlation analysis was applied to assess the relationship between maximal oxygen uptake per kilogram body weight (VO_2_/kg) and other indicators. Multiple linear regression analysis was then performed with maximal VO_2_/kg as the dependent variable to evaluate influencing factors.

**Results:**

Significant differences (P < 0.05) were observed among the three groups in maximal metabolic equivalents (MET), maximal VO_2_/kg, maximal heart rate (HR), fasting plasma glucose (FPG), triglycerides (TG), high-density lipoprotein cholesterol (HDL-C), visceral fat area (VFA), skeletal muscle mass, body fat mass, and obesity index. Age, body mass index (BMI), waist-to-hip ratio, FPG, low-density lipoprotein cholesterol (LDL-C), VFA, skeletal muscle mass, body fat mass, body fat percentage, and obesity index were significantly correlated with maximal VO_2_/kg. Age, BMI, FPG, HDL-C, VFA, and skeletal muscle mass were identified as independent influencing factors for maximal VO_2_/kg, collectively explaining 50.9% of the total variance.

**Conclusion:**

Age, BMI, FPG, HDL-C, VFA, and skeletal muscle mass were influencing factors for maximal VO_2_/kg. VFA was the strongest negative predictor, while skeletal muscle mass served as its positive predictor. These findings may provide a basis for identifying high-risk populations with declining Cardiorespiratory Fitness among individuals with abnormal glucose metabolism.

## 1 Introduction

Diabetes mellitus is one of the most prevalent chronic health conditions worldwide. Prediabetes represents an intermediate metabolic state between normal glucose metabolism and diabetes, encompassing impaired fasting glucose (IFG), impaired glucose tolerance (IGT), and impaired glucose regulation (IGR, IFG + IGT) (1). Poor glycemic control can lead to endothelial dysfunction, inflammation, microvascular injury, myocardial impairment, and skeletal muscle alterations, thereby affecting multiple organ systems. Type 2 diabetes mellitus (T2DM) affects 20%–40% of patients with cardiovascular disease and exacerbates the progression of atherosclerosis, adverse outcomes, and mortality (2).

Cardiorespiratory Fitness (CRF), also referred to as cardiorespiratory fitness, denotes the body’s capacity to uptake, transport, and utilize oxygen during physical activity at a given workload. It is considered one of the most critical indicators of human health (3). Cardiopulmonary exercise testing (CPET) integrates and analyzes continuous dynamic changes in respiratory, circulatory, and metabolic systems during exercise. As the gold standard for evaluating cardiopulmonary metabolic function, CPET has important clinical value in differential diagnosis, prognosis assessment, exercise prescription formulation, and other applications (4). Previous studies have demonstrated (5) that CRF is inversely associated with the risk of diabetes mellitus, and improving CRF may reduce the likelihood of abnormal glucose metabolism.

Body composition refers to the proportional distribution of body components—including water, muscle, fat, and bone minerals—within total body weight. Analysis of body composition not only assists in evaluating physiological normality but also holds screening value for nutrition-related and metabolic disorders (6). Studies have indicated (7) that increased muscle mass and strength may reduce the risk of diabetes, with onset potentially linked to handgrip strength and lean body mass (fat-free mass). Therefore, improving muscle mass and strength should be considered an intervention target for diabetes prevention. However, research investigating the relationship between body composition and CRF in populations with different glucose metabolism statuses remains limited. Previous studies primarily focused on diabetic populations and widely adopted conventional obesity indicators, while insufficient exploration has been conducted on body composition metrics such as visceral fat area and skeletal muscle mass. This study aims to investigate the correlation between CRF and body composition parameters in individuals with different glucose metabolism profiles, thereby providing a basis for formulating and optimizing intervention strategies for CRF-decreased populations.

## 2 Materials and methods

### 2.1 General information

This retrospective study included 144 individuals who voluntarily underwent cardiopulmonary exercise testing at Qinhuangdao First Hospital between January 2023 and June 2025, all of whom were free from T2DM. Based on OGTT results, participants were categorized into the Normal Glucose Tolerance Group (n = 76), Prediabetes Group (n = 22), and T2DM Group (n = 46). The study was approved by the Ethics Committee of Qinhuangdao First Hospital.

### 2.2 Diagnostic criteria

The diagnostic criteria from the China Diabetes Prevention and Treatment Guideline (2020 Edition) were adopted (8).

#### 2.2.1 Diagnostic criteria for diabetes mellitus

Individuals with typical diabetes symptoms (such as polydipsia, polyuria, polyphagia, or unexplained weight loss) meeting any of the following criteria were diagnosed with diabetes:

- Fasting venous plasma glucose ≥ 7.0 mmol/L
- OGTT 2-hour venous plasma glucose ≥ 11.1 mmol/L
- HbA1c ≥ 6.5%
- Random venous plasma glucose ≥ 11.1 mmol/L

If typical symptoms were absent, diagnosis required either two parameters reaching diagnostic thresholds at the same time point or blood glucose values meeting diagnostic criteria at two separate time points (excluding random blood glucose).

#### 2.2.2 Diagnostic criteria for prediabetes

Prediabetes was defined as blood glucose levels higher than normal but below diagnostic thresholds for diabetes. The criteria were:

- Fasting venous plasma glucose ≥ 6.1 mmol/L and < 7.0 mmol/L
- OGTT 2-hour venous plasma glucose ≥ 7.8 mmol/L and < 11.1 mmol/L

### 2.3 Inclusion and exclusion criteria

#### 2.3.1 Inclusion criteria

① Age > 18 years;
② Underwent cardiopulmonary exercise testing;
③ No prior history of diabetes mellitus.

#### 2.3.2 Exclusion criteria

① History of pulmonary embolism, asthma, or chronic lung disease;
② Severe cardiovascular disease (unstable angina, recent myocardial infarction, severe arrhythmia, heart failure, etc.);
③ Severe macrovascular or microvascular complications;
④ Severe hepatic or renal disease;
⑤ Impaired limb mobility;
⑥ Neuromuscular disorders or other contraindications to exercise;
⑦ Participation in professional medical rehabilitation or exercise training within the past six months;
⑧ Use of medications affecting bone metabolism and body composition (glucocorticoids, estrogen, thyroid hormone, parathyroid hormone, calcitonin, bisphosphonates);
⑨ Incomplete clinical data.

### 2.4 Methods

#### 2.4.1 Measurement of basic anthropometric parameters

Patients’ general information was recorded, including age, sex, height, weight, waist circumference, hip circumference, and history of hypertension. Body Mass Index (BMI) and Waist-to-Hip Ratio (WHR) were calculated. BMI = weight (kg) ÷ height² (m²); WHR = waist circumference (cm) ÷ hip circumference (cm).

#### 2.4.2 Collection of laboratory data

The following indicators were collected: fasting plasma glucose (FPG), alanine aminotransferase (ALT), aspartate aminotransferase (AST), uric acid (UA), triglycerides (TG), total cholesterol (TC), high-density lipoprotein cholesterol (HDL-C), and low-density lipoprotein cholesterol (LDL-C).

#### 2.4.3 Measurement of visceral fat area and body composition

All subjects underwent measurements of VFA and body composition in the morning after fasting, conducted in a hospital laboratory using a bioelectrical impedance analyzer. Body composition indicators included total muscle mass, fat mass, body fat percentage, and obesity index.

#### 2.4.4 Cardiopulmonary function assessment

This study utilized the CareFusion MasterScreen CPX Exercise Cardiopulmonary Testing System, with the accompanying software being Jlab To CardioSoft V6.7. The test utilizes the built-in oxygen/carbon dioxide gas analyzer of the instrument to measure exhaled O_2_ and CO_2_ concentrations in real time, calculating core parameters such as VO_2_ and VCO_2_. During testing, subjects breathe ambient air without the use of additional mixed gases. The gas analyzer is calibrated using standard calibration gas with the following composition: 5% CO_2_, 16% O_2_, and N_2_ balance. Progressive load exercise tests are performed using a power bicycle (power pedal). The testing procedure strictly adheres to system standards. Subjects are only tested after all calibrations are confirmed to be compliant. CPET was conducted by certified professionals. Gas exchange parameters, such as oxygen consumption, were monitored continuously during exercise, while heart rate was recorded simultaneously.

VO_2_max was defined as the oxygen uptake plateau, persisting for more than 30 seconds, when oxygen consumption no longer increased despite escalating exercise intensity. The anaerobic threshold (AT) was defined as the oxygen uptake (VO_2_) during exercise at which aerobic metabolism did not yet require supplementation from anaerobic metabolism, representing the highest VO_2_ before lactic acidosis onset. The AT was determined using the V-slope method, with the inflection point at which the slope of the carbon dioxide output curve diverged from that of the oxygen uptake curve serving as the reference position.

The following parameters were recorded at VO_2_max and AT for each group: metabolic equivalents (MET), VO_2_, oxygen uptake per kilogram body weight (VO_2_/kg), systolic blood pressure, diastolic blood pressure, heart rate (HR), and 1-minute post-exercise recovery heart rate.The test was terminated when the following criteria were met: exercise duration exceeding 6 minutes, attainment of an oxygen uptake plateau maintained for at least 30 seconds, and achievement of other target requirements such as AT.

#### 2.4.5 Statistical analysis

Statistical analyses were performed using IBM SPSS Statistics, Version 25.0 (IBM Corp., Armonk, NY, USA). Normally distributed data are presented as mean ± standard deviation (x̄ ± s). Differences among the three groups were analyzed using analysis of variance (ANOVA). Non-normally distributed data are expressed as median (P25, P75) [M (P25, P75)], with differences assessed using non-parametric tests. Univariate correlation analysis was performed to examine the relationship between Cardiorespiratory Fitness indicators and other parameters. Multiple linear regression analysis was applied to identify factors influencing Cardiorespiratory Fitness indicators. A two-sided P-value <0.05 was considered statistically significant.

## 3 Results

### 3.1 Comparison of general characteristics, laboratory parameters, and cardiorespiratory fitness among the three groups

No significant differences (P ≥ 0.05) were observed among the Normal Glucose Tolerance Group, Prediabetes Group, and Diabetes Mellitus Group with respect to sex, age, height, anaerobic threshold MET, anaerobic threshold VO_2_, anaerobic threshold VO_2_/kg, anaerobic threshold HR, anaerobic threshold systolic pressure, anaerobic threshold diastolic pressure, maximal VO_2_, maximal systolic pressure, maximal diastolic pressure, TC, LDL-C, UA, AST, ALT, and body fat percentage. Significant differences (P < 0.05) were observed in weight, BMI, waist circumference, hip circumference, waist-to-hip ratio, maximal METs, maximal VO_2_/kg, maximal HR, FPG, TG, HDL-C, VFA, skeletal muscle, body fat mass, and obesity index. See **Table 1**.

**Table 1:** Comparison of general characteristics, laboratory indicators, and primary cardiopulmonary endurance parameters.

Group	Normal glucose tolerance group	Prediabetes group	Diabetes mellitus group	F/Z/χ2	p
n	76	22	46	--	
Sex (female)	42	12	17	4.127	0.127
Age	55.32 ± 11.11	56.82 ± 9.56	59.96 ± 8.40	3.039	0.051
Height	162.31 ± 7.39	162.38 ± 6.73	164.74 ± 7.95	1.622	0.201
Weight	66.64 ± 8.30	71.87 ± 10.11^#^	73.07 ± 11.85^#^	6.970	0.001
BMI	25.32 ± 2.85	27.23 ± 3.35^#^	26.85 ± 3.50^#^	5.075	0.007
Waist circumference	88.93 ± 8.11	94.33 ± 8.43^#^	95.46 ± 9.89^#^	8.978	<0.001
Hip circumference	95.57 ± 3.95	98.44 ± 5.53^#^	97.72 ± 5.74^#^	4.531	0.012
WHR	0.93 ± 0.06	0.96 ± 0.04	0.98 ± 0.07^#^	9.399	<0.001
FPG	5.24 (4.90, 5.53)	6.14(5.18, 6.41)	7.05(6.16, 8.61)	96.350	<0.001
TG	1.25 (0.87, 1.70)	1.40(1.06, 2.10)	1.70(1.13, 2.52)	11.861	0.003
TC	4.89 ± 0.96	4.99 ± 0.90	4.87 ± 1.12	0.102	0.903
HDL-C	1.19 ± 0.33	1.06 ± 0.28	0.94 ± 0.26^#^	9.010	<0.001
LDL-C	2.63 ± 0.77	2.84 ± 0.61	2.75 ± 0.70	0.816	0.445
UA	303.65 ± 78.54	342.96 ± 105.51	330.39 ± 109.98	2.069	0.130
ALT	17.3 (13.0, 24.5)	23.1(15.05, 31.9)	20.2(14.25, 32.55)	3.330	0.189
AST	20.0 (16.2, 23.5)	21.8(18.85, 27.00)	19.7(16.55, 24.7)	0.214	0.898
Anaerobic Threshold MET	4.09 ± 0.99	3.76 ± 0.56	3.85 ± 0.61	1.986	0.141
Anaerobic Threshold VO_2_	1093.00 (878.22, 1221.75)	1177.00(980.48, 1356.50)	1034.00(888.74, 1126.75)	2.528	0.283
Anaerobic Threshold VO_2_/kg	14.7 (12.9, 17.9)	15.6(13.27, 18.3)	14.3(12.05, 16.35)	3.271	0.195
Anaerobic Threshold HR	110.96 ± 14.96	114.57 ± 15.44	108.05 ± 11.03	1.697	0.187
Anaerobic Threshold Systolic Blood Pressure	145.83 ± 22.33	150.05 ± 23.30	153.88 ± 17.40	2.126	0.123
Anaerobic Threshold Diastolic Blood Pressure	79.13 ± 11.94	79.46 ± 13.92	78.61 ± 8.21	0.051	0.951
maximal MET	5.71 ± 1.37	5.12 ± 0.87	5.05 ± 0.91^#^	5.299	0.006
maximal VO_2_	1332.97 ± 362.44	1295.83 ± 335.52	1293.50 ± 305.85	0.231	0.794
maximal VO_2_/kg	20.00 ± 4.77	17.89 ± 3.03^#^	17.69 ± 3.17^#^	5.429	0.005
maximal HR	132.61 ± 16.33	139.91 ± 19.14	123.98 ± 18.30^#&^	6.939	0.001
maximal Systolic Blood Pressure	175.99 ± 26.14	179.50 ± 24.48	180.57 ± 31.77	0.426	0.654
maximal Diastolic Blood Pressure	81.76 ± 13.58	84.73 ± 12.27	78.48 ± 10.42	2.057	0.132
VFA	102.36 ± 35.97	124.55 ± 36.12^#^	113.35 ± 37.21	3.599	0.030
Skeletal muscle	25.19 ± 4.21	25.84 ± 4.30	27.90 ± 5.55^#^	4.821	0.009
Body Fat Mass	20.74 ± 5.78	24.84 ± 6.85^#^	22.42 ± 6.50	3.981	0.021
Body Fat Percentage	30.99 ± 7.41	34.35 ± 6.72	30.56 ± 7.26	2.235	0.111
Obesity index	118.21 ± 14.08	126.95 ± 16.14^#^	124.46 ± 15.48^#^	3.934	0.022

^#^ , compared with the normal group, p <0.05; ^&^, compared with the prediabetes group, p <0.05.

BMI, body mass index; WHR, Waist-to-Hip Ratio; FPG, fasting plasma glucose; TG, triglyceride; TC, total cholesterol; HDL-C, high density lipoprotein cholesterol; LDL-C, low density lipoprotein cholesterol; UA, serum uric acid; ALT, alanine aminotransferase; AST, aspartate aminotransferase; MET, metabolic equivalents; VO_2_, oxygen uptake; VO_2_/kg, oxygen uptake per kilogram body weight; HR, maximal heart rate; VFA, visceral fat area.

### 3.2 Correlations between maximal VO_2_/kg and other parameters

**Figure 1:**
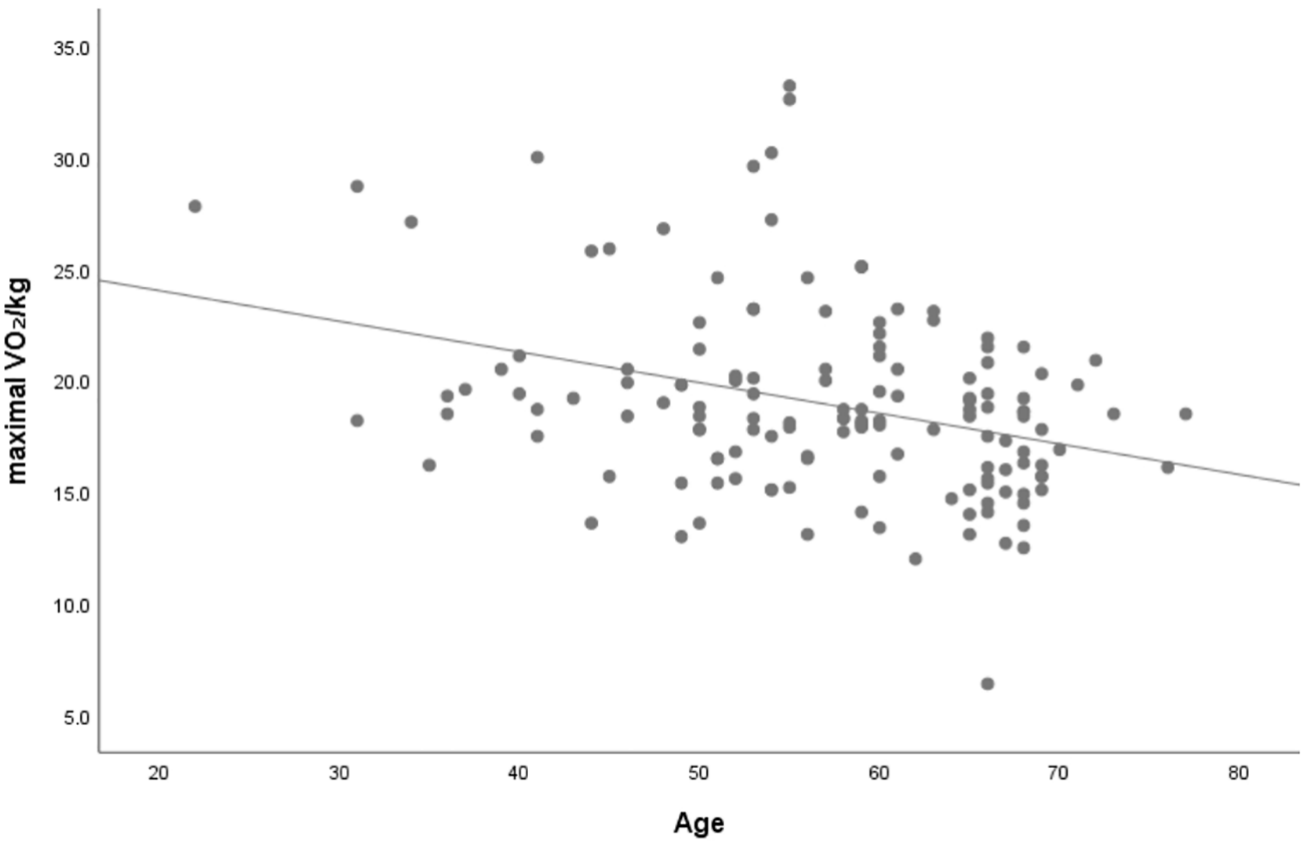
Relationship between age and maximal VO_2_/kg. VO_2_/kg, oxygen uptake per kilogram body weight.

**Figure 2:**
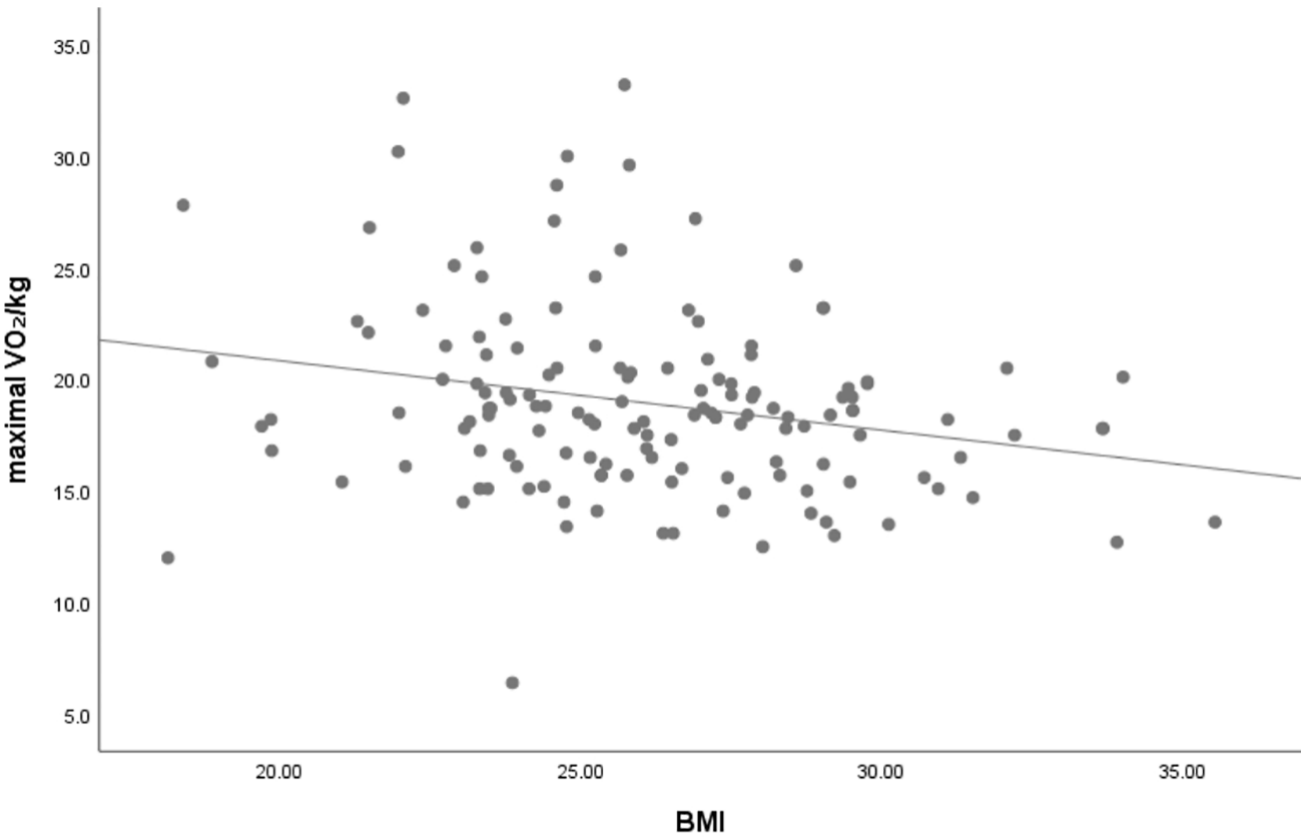
Relationship between BMI and maximal VO_2_/kg. BMI, body mass index; VO_2_/kg, oxygen uptake per kilogram body weight.

**Figure 3:**
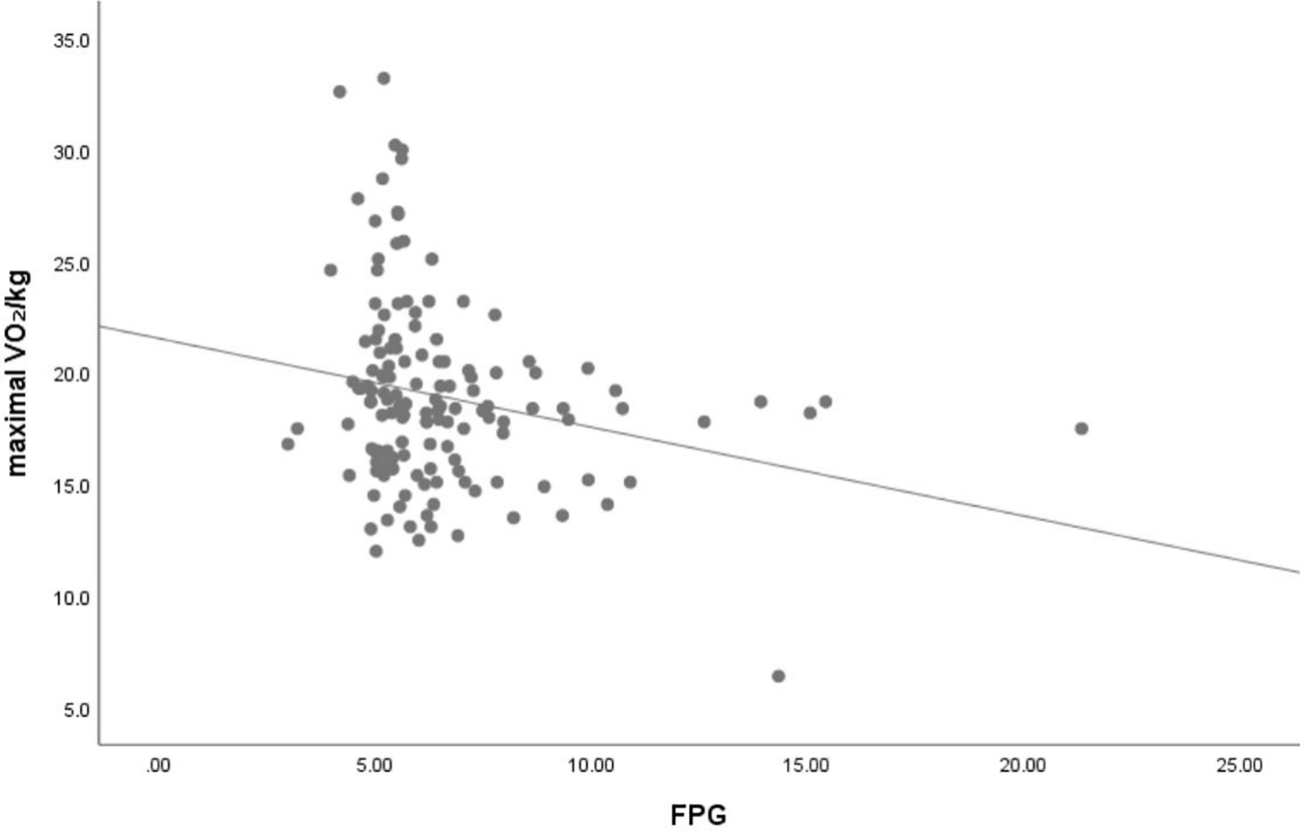
Relationship between FPG and maximal VO_2_/kg. FPG, fasting plasma glucose; VO_2_/kg, oxygen uptake per kilogram body weight.

**Figure 4:**
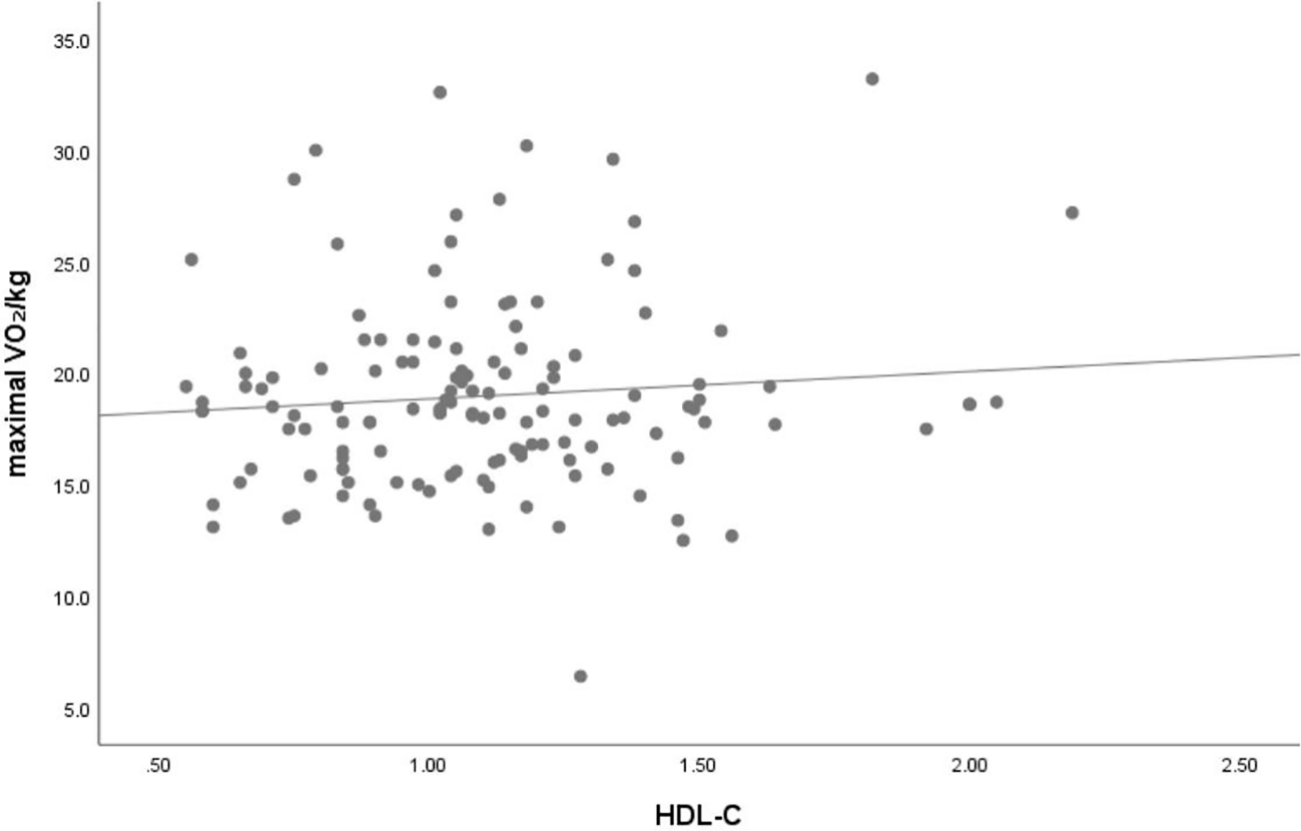
Relationship between HDL-C and maximal VO_2_/kg. HDL-C, high density lipoprotein cholesterol; VO_2_/kg, oxygen uptake per kilogram body weight.

**Figure 5:**
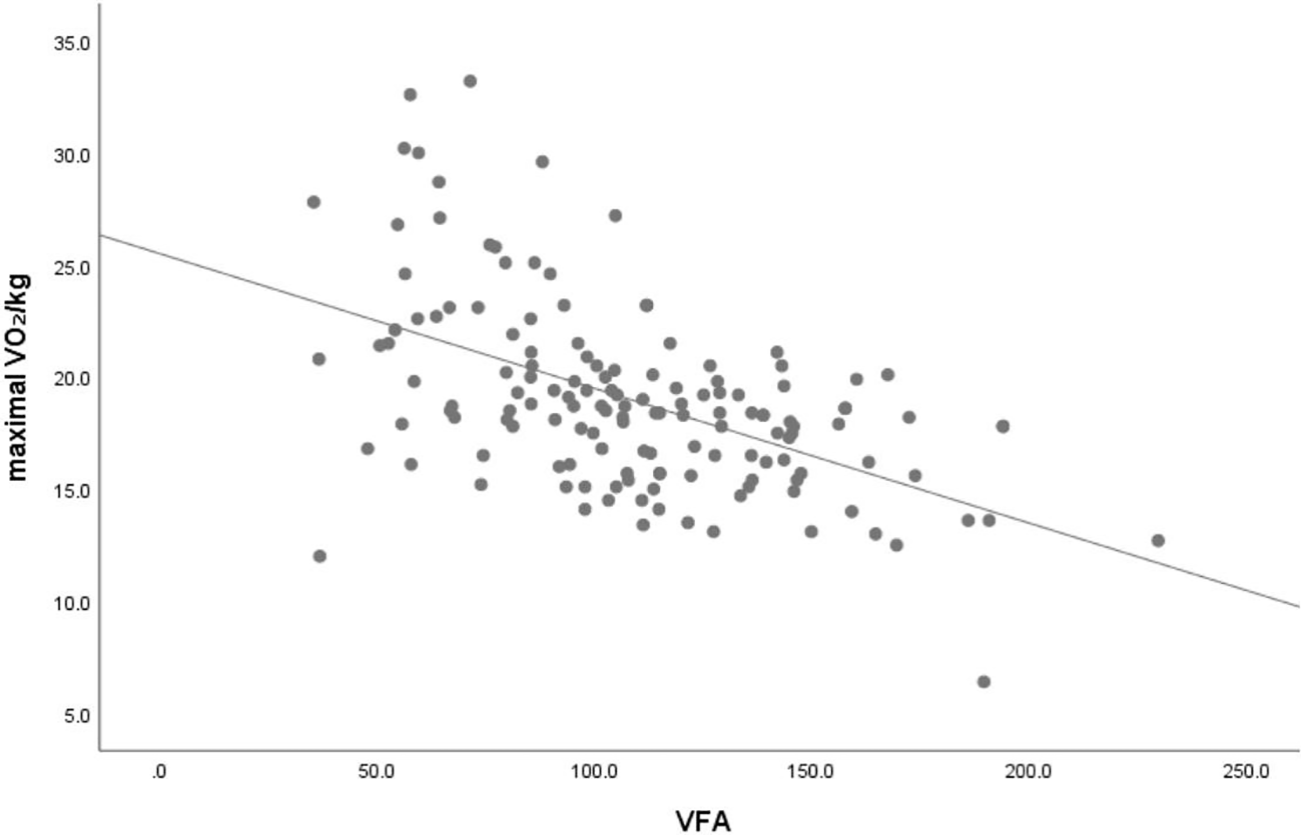
Relationship between VFA and maximal VO_2_/kg. VO_2_/kg, oxygen uptake per kilogram body weight; VFA, visceral fat area.

**Figure 6:**
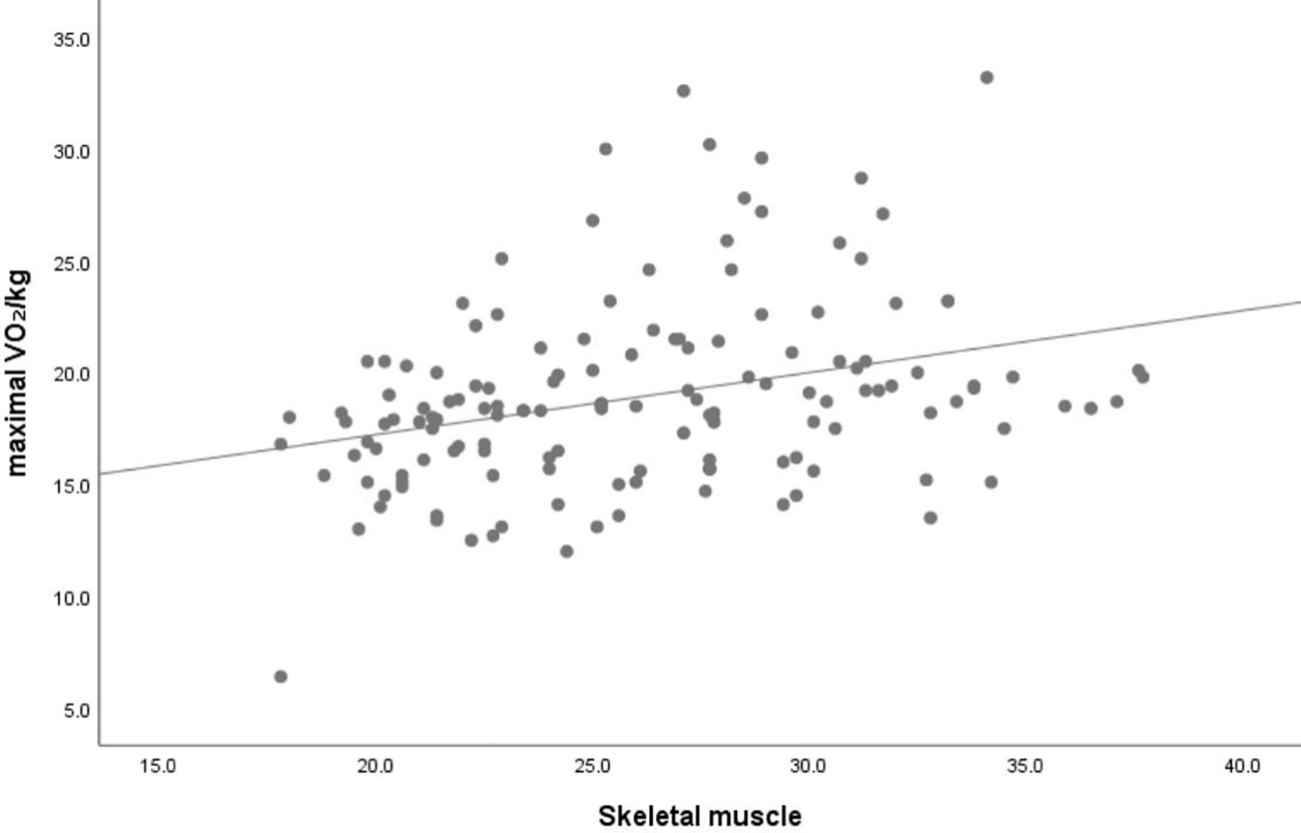
Relationship between skeletal muscle and maximal VO_2_/kg. VO_2_/kg, oxygen uptake per kilogram body weight.

Age, BMI, waist-to-hip ratio, FPG, LDL-C, VFA, skeletal muscle mass, body fat mass, body fat percentage, and obesity index demonstrated significant correlations with maximal VO_2_/kg (P < 0.05). Skeletal muscle mass was positively correlated, whereas all other parameters were negatively correlated with maximal VO_2_/kg. See **Table 2** and **Figures 1**–**6**.

**Table 2:** Correlation between various indicators and maximal VO_2_/kg.

Indicator	r	p
Age	-0.334	<0.001
BMI	-0.239	0.004
WHR	-0.412	<0.001
FPG	-0.213	0.010
TG	-0.075	0.372
TC	-0.099	0.239
HDL-C	0.090	0.281
LDL-C	-0.184	0.028
UA	0.032	0.706
ALT	0.049	0.560
AST	0.118	0.160
VFA	-0.529	<0.001
Skeletal muscle	0.317	<0.001
Body Fat Mass	-0.421	<0.001
Body Fat Percentage	-0.513	<0.001
Obesity index	-0.297	<0.001

BMI, body mass index; WHR, Waist-to-Hip Ratio; FPG, fasting plasma glucose; TG, triglyceride; TC, total cholesterol; HDL-C, high density lipoprotein cholesterol; LDL-C, low density lipoprotein cholesterol; UA, serum uric acid; ALT, alanine aminotransferase; AST, aspartate aminotransferase; VO_2_/kg, oxygen uptake per kilogram body weight; VFA, visceral fat area.

### 3.3 Linear regression analysis of maximal VO_2_/kg and related indicators

Using maximal VO_2_/kg as the dependent variable and age, BMI, waist-to-hip ratio, laboratory indicators, and body composition as independent variables, multiple linear regression analysis with stepwise variable selection was performed. After excluding non-significant variables, age, BMI, FPG, HDL-C, VFA, and skeletal muscle were identified as significant predictors of maximal VO_2_/kg, collectively explaining 50.9% of the variance. See **Table 3**.

**Table 3:** Linear regression analysis of various indicators with maximal VO_2_/kg.

Indicator	β	SE	Beta	t	p
Age	-0.119	0.024	-0.289	-4.908	<0.001
BMI	0.455	0.171	0.350	2.657	0.009
FPG	-0.289	0.109	-0.167	-2.647	0.009
HDL-C	2.650	0.875	0.196	3.030	0.003
VFA	-0.086	0.014	-0.757	-5.925	<0.001
Skeletal muscle	0.165	0.074	0.189	2.230	0.027

R^2^ = 0.529, Adjusted R^2^ = 0.509.

BMI, body mass index; FPG, fasting plasma glucose; HDL-C, high density lipoprotein cholesterol; VFA, visceral fat area.

## 4 Discussion

CRF reflects the body’s ability to transport oxygen from the lungs to skeletal muscle mitochondria for sustained high-intensity physical activity, representing the integrated function of the cardiovascular, pulmonary, and musculoskeletal systems (5). Low CRF is a strong predictor of adverse outcomes in numerous chronic diseases, including T2DM and cardiovascular disease, as well as all-cause mortality (9, 10).

Previous studies have shown (11) that individuals with T2DM exhibit reduced pulmonary function and lower maximal VO_2_/kg compared with healthy controls, which is associated with poor blood glucose control and longer diabetes duration. Even after adjusting for potential confounders (such as physical activity, hypertension, hyperlipidemia, and diabetes duration), a significant negative correlation between T2DM and CRF persisted. This suggests that, independent of other influencing factors, T2DM itself directly impairs CRF (12). Among adolescent males, low CRF was negatively correlated with FPG, whereas no significant association was observed between CRF and FPG in females (13). Zheng et al. (14) reported that middle-aged men with IFG exhibited significantly lower CRF than healthy individuals, characterized by decreased VO_2_max and impaired cardiac function. These findings suggest a link between baseline CRF and the progression of abnormal glucose metabolism.This study found that the maximal VO_2_/kg was significantly reduced in patients with T2DM, while the anaerobic threshold index showed no significant change. This dissociation phenomenon indicates that exercise limitation in diabetic patients primarily stems from impaired myocardial reserve function and decreased oxygen delivery capacity, rather than primary mitochondrial dysfunction (15, 16).

Our results are consistent with these observations, showing a progressive decline in both maximal MET and maximal VO_2_/kg across the Normal Glucose Tolerance Group, Prediabetes Group, and Diabetes Mellitus Group. Furthermore, the Diabetes Mellitus Group demonstrated lower maximal HR compared with both the Normal Glucose Tolerance and Prediabetes Groups. while no statistically significant differences were observed in other parameters of the anaerobic threshold.

Skeletal muscle plays a crucial role in maintaining strength, balance, endurance, and mobility. Among patients with T2DM, the incidence of sarcopenia, characterized by the progressive loss of skeletal muscle mass, increases with advancing age and worsening insulin resistance, thereby significantly impairing mobility and elevating the risk of disability (17). Reduced lean body mass is associated with impaired glycemic control in diabetic patients, likely because skeletal muscle serves as the primary site of insulin action. Compared with non-diabetic adults, diabetic patients present with increased fat mass and decreased lean body mass (18). A case-control study reported that individuals with prediabetes had significantly higher body fat percentage and visceral fat percentage compared with those with normal blood glucose, whereas their skeletal muscle percentage was significantly lower (19). The incidence of prediabetes showed a positive correlation with body fat percentage and a negative correlation with skeletal muscle percentage. These findings suggest that significant alterations in body composition occur even in prediabetic individuals, supporting the recommendation that body composition improvement should be incorporated into prediabetes intervention strategies. Excessive fat mass, particularly visceral obesity, exerts multiple detrimental effects on muscle health. Visceral fat may contribute to intramuscular and intermuscular fat infiltration, thereby impairing mitochondrial function, increasing reactive oxygen species production, and stimulating pro-inflammatory cytokine secretion (20). The proliferation of visceral adipose tissue fosters a pro-inflammatory microenvironment, impairing insulin signaling pathways in peripheral tissues. This insulin resistance subsequently suppresses mitochondrial biosynthesis and oxidative phosphorylation capacity in skeletal muscle fibers, thereby establishing a bidirectional pathological interaction between obesity and muscle cell metabolism (21). Luo et al. (22) reported that individuals with T2DM typically exhibit elevated visceral fat levels. VFA was independently and significantly associated with HbA1c in patients with T2DM. Furthermore, an inverted U-shaped relationship was observed between VFA and HbA1c, with an inflection point range of 8.36% to 8.88%.

This study revealed that the Prediabetes Group and the Diabetes Mellitus Group exhibited higher VFA, skeletal muscle mass, body fat mass, and obesity index compared with the Normal Glucose Tolerance Group, although no significant differences in body fat percentage were observed across the three groups.

Both previous research and the present study indicate significant associations between Diabetes Mellitus and CRF, as well as between Diabetes Mellitus and body composition indicators. This raises the question of whether associations also exist between CRF and body composition indicators. Our findings demonstrate significant correlations between maximal VO_2_/kg and age, BMI, waist-to-hip ratio, FPG, LDL-C, VFA, skeletal muscle, body fat mass, body fat percentage, and obesity index. Among these variables, skeletal muscle showed a positive correlation with maximal VO_2_/kg, whereas all other indicators showed negative correlations. Furthermore, multiple linear regression analysis revealed that age, BMI, FPG, HDL-C, VFA, and skeletal muscle collectively served as determinants influencing maximal VO_2_/kg, accounting for 50.9% of the total variance.

Previous research (14) has reported significant negative correlations between maximal VO_2_ and BMI, body fat percentage, and fat mass. In addition, a strong linear relationship has been documented between maximal VO_2_ and skeletal muscle mass (23). A study by Cooper et al. (24) further emphasized the necessity of linking CRF to skeletal muscle mass estimates in adolescents. They observed a strong linear relationship between maximal VO_2_ and lean body mass in both high-BMI and normal-BMI adolescent populations. Our study found that in univariate analysis, BMI was negatively correlated with VO_2_max, but after controlling for other covariates such as visceral fat and skeletal muscle, BMI showed a positive correlation with VO_2_max. This indicates that individuals with higher lean body mass can maintain better oxygen utilization capacity despite having a higher overall weight. Other studies (25) have found that body fat exerts a negative impact on CRF, but this effect is independent of skeletal muscle mass. In adolescents, lower-body muscle strength and CRF show persistent negative correlations with total body fat and central adiposity. Conversely, adolescents with higher levels of central body fat demonstrate superior upper-body muscle strength (26). McInnis et al. (27) demonstrated that athletes with lower body fat percentage exhibited significantly higher VO_2_max compared with males of similar weight but with normal body fat percentage, indicating that muscle mass plays a key role in determining VO_2_max. A Korean study (28) revealed that CRF levels were positively correlated with skeletal muscle mass index and negatively correlated with VFA, suggesting that low CRF may serve as a potential indicator of reduced muscle mass and visceral obesity among Korean adults. Visceral fat accumulation is associated with mitochondrial dysfunction, increased oxidative stress, exacerbated inflammation, insulin resistance, and cardiovascular dysfunction, all of which contribute to reduced VO_2_max (29). Wang et al. (30) investigated the association between metabolic health indicators and CRF by adjusting for covariates related to CRF (age, smoking status, and alcohol consumption). Multiple linear regression analysis revealed that FPG and HDL-C were significantly correlated with maximal VO_2_ in males. However, a study of South African firefighters (31) found no association between VO_2_max and TC, HDL-C, or LDL-C, but identified a negative correlation with TG. After participating in six months of moderate aerobic training, both prediabetic and diabetic patients demonstrated significantly increased VO_2_max, reduced TG and LDL-C levels, and elevated HDL-C levels (32). Studies by Men et al. further indicated that high-intensity interval training improves cardiopulmonary health indicators (VO_2_max and heart rate) as well as blood lipid levels (33). Elevated HDL-C levels may reflect improved endothelial and autonomic nervous system function, thereby enhancing oxygen transport and cardiovascular efficiency (34). Zaki et al. (35) demonstrated a strong positive correlation between heart rate variability indices (particularly RMSSD and HF components) and VO_2_max in individuals with T2DM, suggesting that enhanced parasympathetic modulation accompanies higher fitness levels and metabolic stability.

In summary, controlling body weight, reducing visceral fat area, and increasing skeletal muscle mass contribute to improvements in CRF. By lowering blood lipid and blood glucose levels while enhancing metabolic function, thereby increasing CRF and preventing or delaying the onset and progression of metabolic diseases. Patients with abnormal glucose metabolism should be encouraged to enhance CRF through multiple approaches, including engaging in at least 150 minutes of moderate-intensity exercise weekly. Aerobic exercise should form the core component, supplemented with resistance training to increase muscle mass. Modifying sedentary behavior by incorporating routine physical activities such as walking and stair climbing is also recommended. Regular monitoring of blood glucose, blood lipids, and body composition, along with appropriate weight management, is essential.

This study has several limitations. This study is a retrospective study with a limited sample size, which may introduce residual confounding factors, lack of direct physical activity assessment, and single-center study environment limitations. Further expansion of the sample size for multi-regional, multi-center studies is required to enhance the accuracy of the results and reduce bias. Additionally, factors such as participants’ daily activity levels, dietary habits, underlying diseases, complication, and medication regimens may influence the outcomes, warranting further clinical research exploration.

## Data Availability

The raw data supporting the conclusions of this article will be made available by the authors, without undue reservation.
